# The Scale-Up of a Digital Health Intervention (Healthy Beginnings for HNEKids) Targeting the First 2000 Days: Protocol for a Randomized Controlled Trial

**DOI:** 10.2196/81390

**Published:** 2025-12-05

**Authors:** Alison L Brown, Nayerra Hudson, Jacklyn Jackson, Jessica Pinfold, Luke Wolfenden, Nicole Nathan, Rebecca Sewter, Sienna Kavalec, Lynda Davies, Sarah Young, Hannah McCormick, Sonya Stanley, Tessa Delaney, Christophe Lecathelinais, Paul Craven, Sinead Redman, Emma Cushing, Nguyet de Mello, Karen Lee, Rachel Sutherland

**Affiliations:** 1 Hunter New England Local Health District Newcastle, NSW Australia; 2 School of Medicine and Public Health University of Newcastle Australia Newcastle, NSW Australia; 3 Hunter Medical Research Institute Newcastle, NSW Australia; 4 National Centre of Implementation Science Newcastle, NSW Australia; 5 NSW Ministry of Health Sydney, NSW Australia; 6 eHealth Sydney, NSW Australia; 7 Prevention Research Collaboration, Sydney School of Public Health University of Sydney Sydney, NSW Australia

**Keywords:** digital health, scale-up, child health, infant feeding, first 2000 days

## Abstract

**Background:**

Digital health interventions, delivered directly to parents’ mobile phones, could transform the delivery of health care during the first 2000 days of a child’s life. Healthy Beginnings for Hunter New England Kids (HB4HNEKids) is an innovative SMS text message–based model of care that provides age-and-stage relevant preventative health information to parents during the first 2000 days. While HB4HNEKids demonstrates promise for population-wide scale-up, the optimal method for achieving universal, cost-efficient, and equitable scale-up remains unclear.

**Objective:**

This protocol outlines a randomized controlled trial that evaluates 2 models for scaling up HB4HNEKids: an “opt-in” clinician-initiated model (intervention) and an “opt-out” system-initiated model (control). The trial will assess program reach (the number and proportion of eligible parents receiving HB4HNEKids) and participant representativeness to identify the most effective, equitable, and efficient approach to scaling up care for families during the first 2000 days.

**Methods:**

A randomized controlled trial will be conducted in 6 Child and Family Health Service (CFHS) sectors (39 CFHS units) within the Hunter New England region of New South Wales, Australia. The 6 CFHS sectors will be randomized in a 1:1 ratio to one of 2 arms, stratified by sector location and average number of births per annum. The intervention arm (a clinician-initiated opt-in model of care) will involve a series of implementation support strategies delivered to CFHS staff (ie, training, audit, and feedback) to support clinicians in connecting eligible families to the HB4HNEKids program. The control arm (a system-initiated opt-out model of care) will use existing health service data to identify participants meeting the predefined eligibility criteria to automatically initiate the commencement of HB4HNEKids messages to families (ie, will not require input from CFHS staff). The primary outcome will assess the reach and representativeness of participants receiving HB4HNEKids. Secondary outcomes will include child health behaviors (breastfeeding rates; age of introduction to solids; child fruit, vegetable, and discretionary food intakes; as well as child immunization rates) captured via parent survey, as well as the cost-effectiveness.

**Results:**

This trial commenced in July 2024, and as of July 2025 enrolled 4212 participants. Data collection (via parent survey) commenced in January 2025 and is projected to end in July 2027.

**Conclusions:**

Currently, little is known about the most effective model of scaling up digital health interventions. This trial will generate novel evidence for informing the effective scale-up of evidence-based health promotion programs that aim to provide universal care, which are needed to maximize the potential population health gains.

**Trial Registration:**

Australian New Zealand Clinical Trials Register ACTRN12624000655549p; https://tinyurl.com/4krc9za5

**International Registered Report Identifier (IRRID):**

DERR1-10.2196/81390

## Introduction

The first 2000 days of a child’s life (ie, conception to 5 y of age) represent a critical window for promoting child health and development, and lay the foundations for a child’s lifelong physical, mental, social, and emotional health and well-being [1]. Early exposure to health-promoting behaviors, including breastfeeding, consumption of healthy diets, and adequate physical activity, can have powerful programming effects [2,3]. For example, longer breastfeeding duration is associated with better neurodevelopment outcomes, lower risk of childhood and adulthood obesity, as well as reduced risk of type 2 diabetes [4-7]. Reviews have also found that child physical activity and healthy dietary intakes before age 5 are associated with beneficial cognitive outcomes, motor skill development, and improved measures of adiposity [8].

As lifestyle exposures during the first 2000 days can have implications for children’s academic, social, and health trajectories, there remains a need to support families to achieve optimal health behaviors during the first 2000 days [9]. Despite national infant feeding guidelines recommending that infants are exclusively breastfed until around 6 months of age and breastfeeding continued until 12 months and beyond [10], population data demonstrate that Australian exclusive breastfeeding rates decline from 74% at 2 months to 38% at 6 months, with only 43% of infants receiving any breast milk at 12 months [11,12]. In addition, while 97% of Australian children aged 2-3 years met the recommended intakes for fruit, only 20% of 2 to 3-year-olds met the recommendations for vegetables, with the proportion of children meeting fruit and vegetable recommendations drastically dropping by 4-8 years [13]. The report further identified that the proportion of children who did not usually eat fruit daily has doubled over the last decade (2011-2022), and the proportion of children who do not usually eat vegetables daily has tripled [13]. This reinforces the need to provide parents with direct and consistent support from birth to 5 years, to further limit the prevalence of suboptimal health behaviors during these crucial developmental stages.

For decades, the provision of universal health care during the first 2000 days has been recommended by national and international policies [14-16]. In Australia, this support has been traditionally offered by a range of health care providers via face-to-face models; however, this care can be fragmented and poorly attended by parents. For example, in New South Wales, Australia, Child and Family Health Services (CFHSs) provide access to routine universal care via face-to-face in-home or clinic visits. However, 2024-2025 data from the Hunter New England Local Health District (HNELHD) of New South Wales (internal data) indicates just 83% of parents accept the initial 1 to 4-week health check home visits, a trend that is consistent with wider New South Wales data [17], with engagement steadily declining to approximately 5% by 4 years. While ongoing CFHS usage is associated with reduced risk of hospitalization, emergency visits, and early identification of health and developmental concerns [18], barriers to parent engagement with face-to-face health care remain, including time, travel, and scheduling conflicts [19].

Digital models of health care delivery may address issues of reach and engagement by providing asynchronous care that parents can use at their convenience. Mobile health (mHealth) interventions (ie, SMS text messages), delivered directly to parents’ or carers’ mobile phones, could greatly enhance the delivery of health care during the first 2000 days [20]. This notion is supported by a meta-review including 25 systematic reviews that demonstrated parent-targeted mHealth interventions can improve literacy, antenatal service usage, child vaccination rates, preventive health behaviors, and maternal health outcomes [21]. As such, digital models of care that support families across the first 2000 days are recommended to improve child health outcomes [20].

Healthy Beginnings for Hunter New England Kids (HB4HNEKids) is an innovative SMS text message (mHealth) program designed to be delivered alongside the usual CFHS. It provides age-and-stage relevant preventative health information, child health check and immunization reminders, as well as information on health screening and support services, directly to parents during the first 2000 days (commencing within days after birth up until 5 years). HB4HNEKids was adapted from an original efficacy trial demonstrating improvements in infant feeding practices and screen time [22], and a pilot effectiveness trial which evaluated its feasibility, acceptability, and effectiveness when embedded into routine health service delivery within 5 diverse CFHS in the Hunter New England region of New South Wales. The pilot trial, implementing HB4HNEKids into routine health service delivery demonstrated that HB4HNEKids digital model of care program was feasibly delivered to families (delivered to over 6000 parents), reached approximately 70% (6243/8501) of eligible parents at a relatively low cost (approximately Aus $4.66/family per year, equivalent to US $3.03/family per year), had high parental engagement (73/96, 76% of parents or carers reported reading the messages) and acceptability (89/96, 93% of parents liked the program) [23]. The pilot also indicated that HB4HNEKids participants had improved child vegetable intakes (*P*=.006) and parental mental well-being (*P*<.001) at 12 months compared with families who had not received the program [24]. In addition, breastfeeding rates were 5% higher in the HB4HNEKids participants at 6 and 12 months compared with families who had not received the intervention. Thus, while the pilot of the HB4HNEKids program demonstrates promise for population-wide scale-up, the optimal method for achieving universal, cost-efficient, and equitable scale-up remains unclear.

The pilot implementation study of the HB4HNEKids SMS text message program required clinicians to offer the program to families during their first contact with the CFHS (clinician-initiated model). Clinicians provide child health and development assistance and advice, and, where needed, refer patients to secondary services. Clinician-initiated onboarding may increase the salience of the offer of the HB4HNEKids program, resulting in increased engagement and improved impact on behavioral outcomes, particularly as CFHS is considered a trustworthy source for infant and child health information and support [25]. However, engagement with CFHS is fragmented, with only 83% (8638/10393) of families in 2022-2023 attending their 1- to 4-week health check and a significant reduction in use of services thereafter. Additionally, there are barriers that impede a clinician’s ability to enroll participants into digital health interventions (DHIs) like HB4HNEKids, such as a lack of training and education, a lack of time, and a concern for increased workload, competing priorities, as well as infrastructure and technological barriers [26]. A system-initiated model, relying upon an opt-out approach, whereby parents and families are automatically enrolled into digital health programs such as HB4HNEKids, via data and systems, may have the potential to facilitate the universal offering of DHIs to eligible parents, likely at much lower financial cost than developing implementation strategies that address clinician barriers to enrolling participants. Opt-out approaches have shown promise in increasing participation in DHIs internationally [27]. For instance, a study evaluating a maternal and child health SMS text message program in a study from New Zealand found that transitioning from an opt-in to an opt-out model resulted in an increase in enrollments but had no significant effect on withdrawal rates [27]. However, the retrospective study did not report on the acceptability of the opt-out model, nor its impact on behavioral outcomes or sustainability, highlighting the need for further research in understanding factors that influence opt-out approaches. While an opt-out approach may seem promising, offering a DHI that does not include clinician contact may result in less trustworthiness and salience of information, resulting in less engagement in the DHI [28,29]. The effects of such models may also differ by participant characteristics that have the potential to drive inequity [30]. Although opt-out approaches have demonstrated potential in enhancing the reach of DHIs, there is limited data comparing the effects of a clinician-initiated and system-initiated models of care on parent engagement, representativeness, retention, effectiveness, and cost-effectiveness in digital health programs. Therefore, it is necessary to understand the impact of both scale-up models on these outcomes to optimize the success of DHIs.

To address this gap, we aim to conduct a randomized controlled trial (RCT) to test the effects of a clinician-initiated (opt-in) model relative to a system-initiated (opt-out) model (control) of enrolling parents into the HB4HNEKids program. This RCT will explore the impact of the 2 scale-up models on (1) program reach (number and proportion of eligible parents that receive HB4HNEKids) and (2) representativeness (similarity of the characteristics of the parents receiving HB4HNEKids from all parents eligible to do so over the study period). Additionally, we will assess the effects on child health behaviors, costs, as well as implementation and process measures.

## Methods

### Study Design and Setting

An RCT will be conducted in 6 CFHS sectors (made up of 39 CFHS units) in the Hunter New England region of New South Wales, Australia. CFHS receives referrals to provide care to families with children aged 0-5 years and their families (parents or carers) from all public and private maternity services in HNELHD, following birth. In any given year in HNELHD, approximately 10,000 births occur, which is therefore indicative of the number of referrals received by CFHS. CFHS within HNELHD provides care to families from varying sociodemographic backgrounds, with 39% of services located in metro and 61% in regional or rural areas. Approximately 20.6% of babies born in the HNELHD are Aboriginal or Torres Strait Islander, and many families come from culturally and linguistically diverse backgrounds [31].

The research will be conducted and reported in accordance with the requirements of the CONSORT (Consolidated Standards of Reporting Trials) 2010 Statement and the Standards for Reporting Implementation Studies statement 2017 [32]. CFHS sectors will be randomized to one of 2 arms: the intervention arm, a clinician-initiated model, in which participants voluntarily “opt-in” to receive HB4HNEKids, or the control, a system-initiated model, where participants are automatically enrolled based on key eligibility criteria and must “opt-out” if they do not wish to receive the program. The protocol is reported according to the Standard Protocol Items: Recommendations for Interventional Trials (Multimedia Appendix 1) [33]. Any modifications or deviations from the methods outlined within this protocol will be documented by the research or project team, and updates will be logged in our online trial registration and disclosed within any relevant publications. The senior author (R Sutherland), together with the broader research and project team, will oversee the project dissemination plan, including all publications and reports to stakeholders. Authorship will conform to the International Committee of Medical Journal Editors guidelines.

### Ethical Considerations

Approval to conduct this study has been obtained from Hunter New England Research Ethics Committee (2022/ETH00359 and 2023/ETH02782) and the University of Newcastle (H-2022-0129 and R-2024-0095). This research study was granted a waiver to randomize participants and deliver the program as part of usual health service delivery. All participants were regularly sent a link to opt out of the program (n=6 in the first 12 months and n=4 from 1 to 2 years). The opt-out link remains open for participants to access at any stage. For the evaluation of child health behaviors, consent was expressed by the completion of a survey. Given that participant burden to complete surveys to evaluate child health behaviors was minimal (less than 10 min per survey) per data collection time-point, upon survey completion, participants could enter a draw to receive 1 of up to 3 gift cards to the value of Aus $30 (equivalent to approximately US $20) as an evidence-based strategy to encourage participant sustainment and engagement with evaluation activities. Health service and participant data were collected and securely stored using the Research Electronic Data capture (REDCap, version 14.1.2, 2025 Vanderbilt University) tool, hosted by HNELHD, which is a secure research electronic database commonly used in health service research, compliant with health service privacy and confidentiality policies. Data will be anonymized, deidentified, and stored so that only authorized individuals have access to the data.

### Sample and Participants

#### Recruitment

##### CFHSs

In total, 6 of the 7 regions, which take in 39 CFHSs (ie, clinics) of HNELHD, will participate in the scale-up of HB4HNEKids. One region was involved in the HB4HNEKids pilot implementation study over the past 3 years and will therefore be excluded from this study analysis.

##### Parents

The mHealth SMS text message program, HB4HNEKids, will be delivered to eligible parents or carers (from herein named parents) alongside usual face-to-face CFHS care. Parent eligibility criteria for both the intervention and control arms were developed in partnership with CFHS clinical leads, and include: (1) must be eligible to access CFHS located in the HNELHD; (2) must own a mobile phone with SMS capability (96%) [34]; (3) must have a baby who has been discharged from a hospital in the last 8 weeks; (4) had a length of hospital stay less than 45 days; (5) absence of complex social issues resulting in the infant needing out of home care; and (6) baby was born ≥32 weeks of gestation. Furthermore, CFHS clinicians can use their clinical judgment to exclude families from the program at any time based on unforeseen personal circumstances. Over an 18-month intervention period, it is expected that approximately 15,000 parents (ie, 10,000 per year) who are referred to CFHS will be exposed to HB4HNEKids. All eligible parents who are referred to CFHS will be included in the sampling frame and analysis. While this RCT is planned to 24-month follow-up, there is scope to longitudinally follow this cohort across the first 2000 days (until their child is 5 years of age) to explore several outcomes of interest.

### Randomization and Blinding

Given that CFHS clinicians may work across multiple CFHS within a region or sector, the randomization for this trial will be conducted at the CFHS sector level to minimize possible contamination across CFHS sites. The 6 CFHS sectors (including 39 CFHS) will be stratified by geographic location and number of births/year (based on whether the number of births is higher or lower than average), and randomized in a 1:1 ratio to one of the 2 trial arms (clinician-initiated or system-initiated). Randomization will occur by an independent statistician using Microsoft Excel. CFHS will not be blinded to group allocation; however, the primary trial outcome—reach and representativeness of HB4HNEKids—will be objectively assessed via electronic records of service enrollment.

### HB4HNEKids SMS Text Message Program

HB4HNEKids is an SMS text message program delivered to parents who are accessing CFHS after having a baby. The program aims to provide evidence-based age and stage information to parents across the first 2000 days. The mHealth program is an adapted version of the Healthy Beginnings program evaluated via a 3-armed RCT [35] and demonstrated to have an impact on screen time and infant feeding practices. The optimization process for HB4HNEKids from the original Healthy Beginnings program aimed to enhance its effectiveness on breastfeeding duration and a delayed introduction to solids, as well as seeking to improve other health behaviors identified as a priority by health service executives, clinicians, and relevantly aligned policies and frameworks, such as the First 2000 Days framework [16], New South Wales Healthy Eating and Active Living Strategy [36], and National Digital Health Strategy [20]. Optimization of program format and content occurred via an extensive co-design process involving a multidisciplinary team of CFHS staff, Health Promotion practitioners and Dietitians, as well as partners from Aboriginal and Multicultural Health services, and end users. The process used the Behaviour Change Wheel (BCW) [37] and Theoretical Domains Framework (TDF) [38] to optimize the original program, adapting and developing new program content and materials, as well as maximizing recruitment pathways for participants, based on the local context and implementation infrastructure. To minimize resource costs and streamline the model used to provide regular health information to families, the adapted version also sought to use a digital-only format (via SMS text message), rather than providing brochures at key milestones, supplemented by digital care, as detailed in the original efficacy study [35].

The HB4HNEKids program aims to support improved health and developmental outcomes for families by targeting barriers and enablers to optimal preventative health behaviors including (1) breastfeeding and appropriate introduction of solids by providing guidelines and practical information and advice, as well as facilitating referral pathways, to support best-practice infant feeding; (2) child nutrition including guidelines and strategies to implement and address responsive feeding, family foods, reducing discretionary foods, and increasing healthy foods; (3) physical activity and small screen recreation by connecting families to evidence-based resources, credible content, and real-world strategies that facilitate daily activities; (4) child health and development according to age, stage-relevant child milestones, and pathways for more information and support; (5) parental well-being, targeting practical approaches to self-care, connections, and self-efficacy; and (6) immunization and primary health checks by prompting and reminding families about scheduled checks with CFHS or general practitioners.

**Figure 1:**
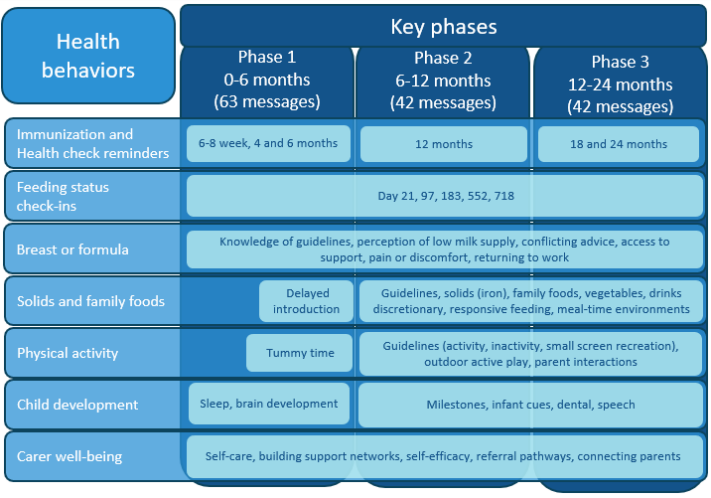
HB4HNEKids message topics by phase.

**Figure 2:**
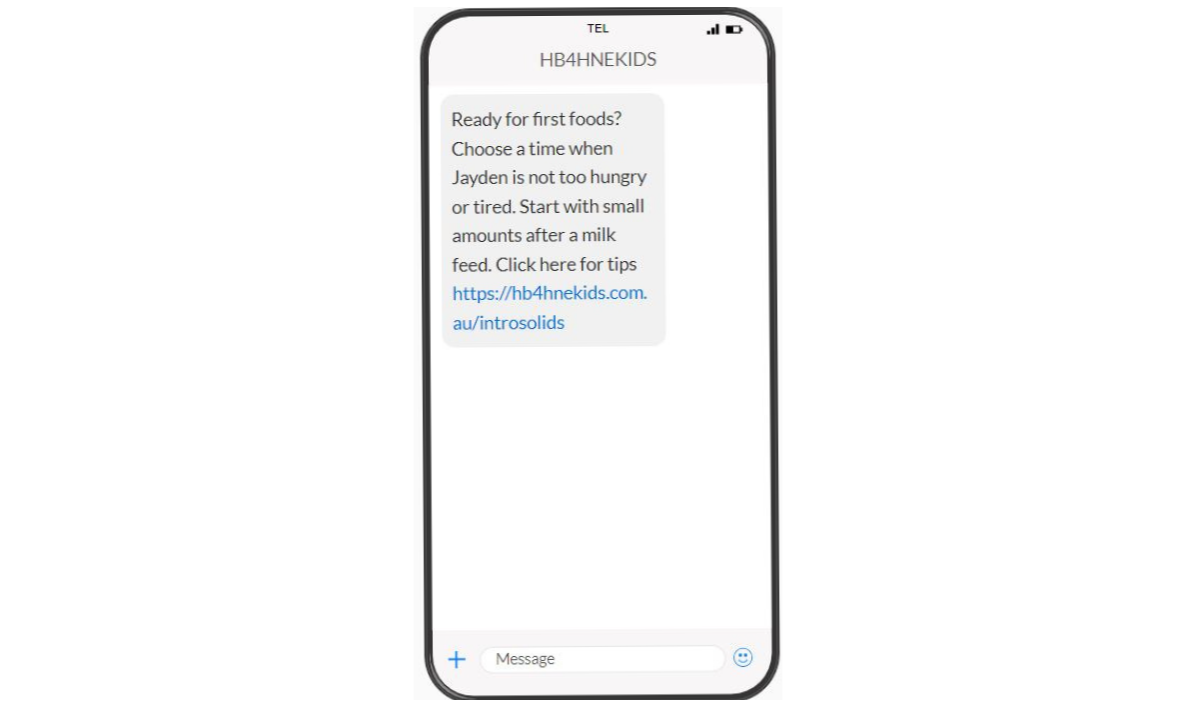
Example SMS text message. HB4HNEKids: Healthy Beginnings for Hunter New England Kids.

SMS text message content was developed as part of a pilot project and optimized to be delivered across both experimental groups in this trial. SMS text messages are tailored to include the infant’s name and infant feeding status (breast, formula, or mixed feeding status). The content includes up to 147 SMS text messages to be delivered over a 24-month period, with more frequent SMS text messages in the first 3 months (approximately twice per week), and every 1-2 weeks for the remainder of the first 2000-day period (Figure 1). The intervention messages included behavior change techniques that were designed to be primarily delivered via SMS text messages. Supplementary content was optional and available to access via embedded links, which require internet access. Almost all SMS text messages included embedded web links to related evidence-based online content, including websites, factsheets, podcasts, and videos (refer to Figure 2 for an example message).

The scheduling of messages was based on the baby’s date of birth (or adjusted date of birth for the first 12 months for babies born prematurely), and daily message outputs were generated using SAS statistical software. A member of the research team generates and uploads daily message outputs to a cloud-based SMS provider, and messages are scheduled to be delivered at varied times between 9 AM and 12 PM, based on expert advice to encourage ongoing engagement with messages. The cloud-based SMS provider (DirectSMS) did not have the capability to track read receipts.

### Intervention

#### Scale-Up Model Development

The intervention design for scale-up was based on findings from the pilot program and an expert task group, established to support the co-design process. A multidisciplinary team of public health practitioners, CFHS staff, service managers, and academics, with expertise in community health services, quality improvement initiatives, stakeholder engagement, behavior change, and implementation science, supported the review of evidence and provided local context to inform co-design of a scale-up model, resulting in a clinician-initiated model versus a system-initiated control model (usual care control).

To identify implementation strategies, barriers and facilitators to digital health care were identified via literature review [26] and findings from the pilot program, which were mapped to behavior change techniques [39] using the BCW and TDF [38]. The Expert Recommendations for Implementing Change (ERIC) [40] was used to define the implementation strategies, and objectives for each strategy were outlined using the Action, Actor, Context, Target, Time (AACTT) framework [41] (Multimedia Appendix 2 [26,38-41]).

#### Preimplementation Strategies

Preimplementation strategies for both the intervention and control arms were used before the trial commenced. These strategies provided CFHS staff members within the Hunter New England region with information on the HB4HNEKids program and provided scope and context for the scale-up of the program. Co-design with CFHS, service managers, and public health practitioners was undertaken to determine what strategies would be useful in preparing CFHS for the HB4HNEKids program throughout the local health district.

The preimplementation strategies, as illustrated in (Multimedia Appendix 2 [26,38-41]), were consistent across the intervention and control and included:

- Involve executive boards: executive endorsement or management support.
- Conduct educational outreach visits and assess for readiness and identify barriers and facilitators: sector-based information sessions, providing information on the pilot study and the proposed future of the program.
- Build a coalition: establishment of sector-based key contact.
- Make training dynamic: brief prerecorded (15 min) presentation for service staff, outlining the program and objectives of the scale-up trial and professional taster messages, which provided clinicians the opportunity to experience a sample of messages (n=13) that families receive during the first 6 months of the program.

#### Intervention: Clinician-Initiated (Opt-In) Model

The clinician-initiated (opt-in) model is based on the process used during the pilot program to support clinicians in connecting families to the program. As per existing health service procedures, CFHS receives a referral from public and private maternity services following the birth of a baby and discharge of the mother and child from the hospital [42]. Upon discharge, CFHS staff contact the parent (participants) via phone call or SMS text message to offer a 1 to 4-week health and development check. During this contact with parents, staff from one of the clinician-initiated CFHS offer HB4HNEKids as part of routine care. CFHS staff also confirm feeding status (ie, if the child was breastfeeding, mixed feeding, or formula feeding), child name, mother's mobile number, and “offer of service” (ie, yes, client refused, or unable to contact after multiple attempts). If a staff member is unable to determine the infant’s feeding status during the initial contact, they will locate the “infant feeding status” via the hospital discharge summary provided by maternity services.

Building on the pilot, a series of additional strategies to improve the process of enrolling parents to the HB4HNEKids program have been co-designed by an expert task group and are outlined in the logic model (Multimedia Appendix 2 [26,38-41]). Following the process used for the intervention design, the established task group used the literature and local expertise to identify and map barriers and enablers to implement a clinician-initiated model. The BCW and TDF were also used to develop implementation strategies [38]; strategies were mapped to the ERIC strategies [40] and defined using the AACTT framework [41].

#### Control: System-Initiated (Opt-Out) Model

The control was a system-initiated (opt-out) model. The selection of a system-initiated model was based on clinician feedback during the pilot phase and automates the connection of families to the program. This model requires minimal input from clinicians. As per existing health service procedures, health service data are generated when CFHS receives a discharge referral from public and private maternity services for a mother or baby following the birth of a baby to initiate usual care via CFHS. This health service data will be used to identify parents who meet the predefined eligibility criteria for the HB4HNEKids program and will also be used to initiate the commencement of program messages, resulting in families automatically receiving the program. Predefined eligibility criteria were developed with and approved by CFHS clinicians. Nonetheless, CFHS staff are encouraged to refer to the program content as a potential trigger for discussions when conducting usual care. As part of this model, clinicians may opt out families from the program if it is not considered appropriate. Families can also opt out themselves, using the established pathways that cease program delivery to a family (eg, via links provided in messages, via links on the program website, or directly through a request to CFHS staff).

### Data Collection Procedures and Measures: Primary Outcome

The primary trial outcome will be the reach and representativeness of the HB4HNEKids program at 4, 6, 12, and 18 months from the commencement of the scale-up phase. The reach of the program by the scale-up model (clinician-initiated vs system-initiated) will be explored. Reach will be defined as: the number of eligible parents that are receiving HB4HNEKids (assessed via project records) and the proportion of parents receiving the program, calculated by the number of eligible parents receiving HB4HNEKids (numerator), divided by the total number of parents eligible for the program (denominator), identified via electronic health service records.

Representativeness will be assessed by comparing the characteristics of the parents receiving HB4HNEKids via each scale-up model to those of the total sample of eligible parents or carers referred to CFHS during the 18-month trial period. Characteristics of eligible parents (collected via electronic health service records and “The Good for Kids Parent Collective”) include parental age, geographic location, highest level of education, employment status (before birth of child), first baby (yes or no), number of children, language spoken at home other than English, and Aboriginal and Torres Strait Islander status. Sample representativeness of parents receiving the HB4HNEKids program at baseline and those remaining engaged at 18 months from both scale-up models will be compared, as well as compared with characteristics representative of the wider Hunter New England population.

### Secondary Outcomes

#### Child Health Behaviors

The effectiveness of HB4HNEKids on key preventative health behaviors including proportion of any breastfeeding at 4, 6, and 12 months old; age of introduction to solids; mean daily serves of vegetables, fruit, and discretionary foods at 12 and 24 months old; and child immunization rates will be assessed using validated items via computer assisted telephone surveys or online surveys (as part of the GFK Parent Collective or using service delivery data) sent out to program participants via a link. The data collected as part of the GFK Parent Collective will not be included within the individual child electronic medical records; however, the data will support the evaluation of this trial and support future health service planning through the ongoing development of the program and informing New South Wales Health services (eg, CFHS).

#### Cost-Effectiveness

The costs, cost-effectiveness, and potential cost savings of the scale-up models will be undertaken from the health service perspective. Resources used for the clinician-initiated and system-initiated models will be assessed via project records (infrastructure, staff, and consumables). Direct costs include digital infrastructure and text messaging platform, labor (health promotion team and CFHS), cost to develop additional material, clinician training, and implementation support. The cost-effectiveness of the scale-up models will be compared with the primary cost-effectiveness outcome, being incremental cost per unit change (%) in reach.

#### Process and Implementation Outcomes

##### Engagement (intervention adoption)

Project records obtained from project databases (REDCap) and the text message platform will be used to assess parental engagement with HB4HNEKids. Engagement data will include the proportion of eligible parents who click through on external hyperlinks embedded into SMS text messages at 4, 6, 12, and 24 months post baby’s birth. Engagement will continue to be measured across the first 2000-day period and reported as part of a further phase of data collection.

##### Implementation

As per Proctor et al [43], the following digital health implementation outcomes will be measured: clinician acceptability, feasibility, and appropriateness using validated items sent to clinicians via a hyperlink to an online survey. Parent acceptability will be assessed using the same validated items embedded within the above-mentioned computer-assisted telephone interviewing or online survey.

##### Maintenance

The parental opt-out rates from HB4HNEKids for intervention and control will be assessed via project and electronic health service records used to deliver the program as per the primary outcome – reach. Characteristics of parents opting out of receiving HB4HNEKids in both groups will also be assessed at 18 months post baby’s birth.

#### Sample Size and Power Calculation

For the primary outcome, a sample of 1126 participants or families per trial arm will enable a detectable difference of 9.8%, assuming the opt-in model will achieve approximately 65% reach at 18 months (based on previous pilot data [awaiting publication]) with 80% power, α of .05, and an intraclass correlation coefficient of 0.05. Given the sectors involved in the trial are estimated to account for 55% of births within the Hunter New England area, achieving this sample should be possible.

### Analysis

Analyses of trial outcomes will be undertaken under an intention-to-treat framework. For assessment of reach of HB4HNEKids—the primary trial outcome—between-group differences will be assessed using mixed logistic regression to account for any potential sector–level clustering effects. The regression model will include fixed effects for each arm of the trial and control for covariates prognostic of the outcome, including participant geographical location and ethnicity. To assess the impact of HB4HNEKids on child health behaviors, we will compare these outcomes between groups (clinician-initiated model vs system-initiated model) using mixed logistic and linear regression models. The model will include fixed effects for service and parent demographics that are found to be significantly different between scale-up models to account for potential consent bias. Subgroup analyses based on parent characteristics will also be conducted to examine differential effects on models of care against the outcome. We will use descriptive and comparative statistics to describe implementation and process outcomes, including engagement, acceptability, feasibility, appropriateness, and maintenance. All statistical tests will be 2-tailed with an α of .05.

Given that the trial is exploring the implications of the scale-up model (not the HB4HNEKids service), a data monitoring committee is not required for this trial. Data will be stored securely in accordance with the requirements of all ethics committees. Data will only be accessible to investigators listed on the ethics application and statisticians.

## Results

**Figure 3:**
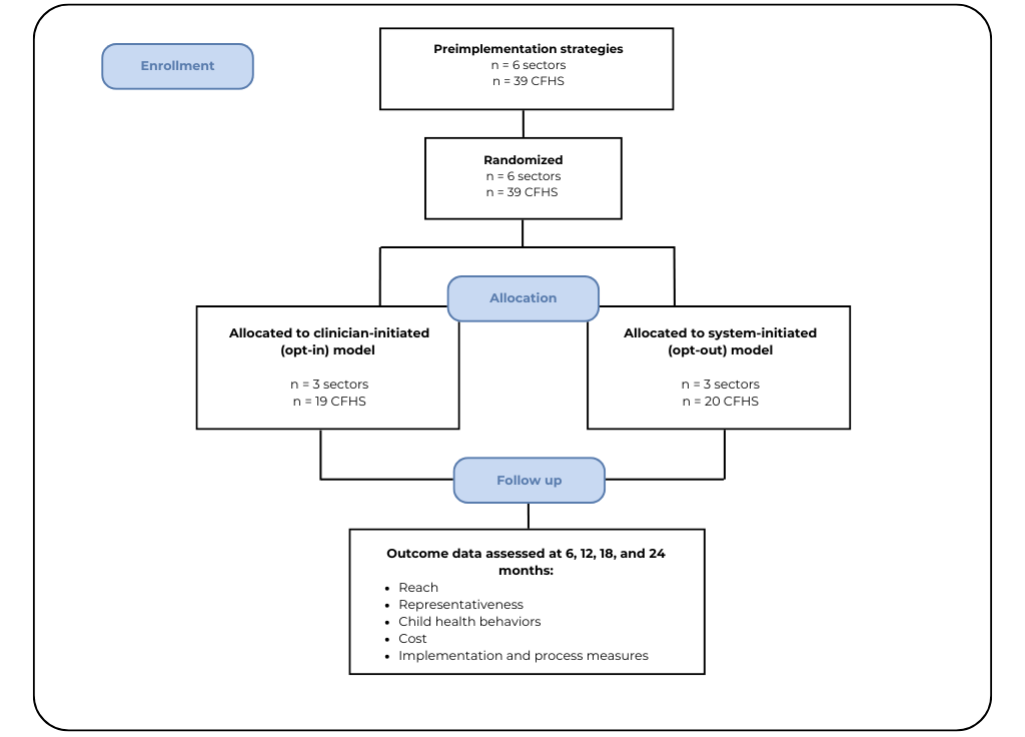
CONSORT flow diagram. CFHS: Child and Family Health Service.

This trial commenced in July 2024, and as of July 2025, enrolled 4212 participants. Data collection (via parent survey) commenced in January 2025 and is projected to end in July 2027 (Table 1). The CONSORT flow diagram of the included CFHS in Hunter New England is presented in Figure 3. Results are expected to be published in 2028 following the completion of data collection.

**Table 1:** Project timeline.

	2024		2025				2026				2027			
	Q3^a^	Q4^b^	Q1^c^	Q2^d^	Q3	Q4	Q1	Q2	Q3	Q4	Q1	Q2	Q3	Q4
Preimplementation strategies – all services	✓													
Implementation strategies – clinician-initiated services only	✓													
Participant recruitment		✓	✓	✓	✓	✓								
Intervention delivery		✓	✓	✓	✓	✓	✓	✓	✓	✓	✓	✓	✓	
6-month data collection^e^				✓	✓	✓								
12-month data collection^e^						✓	✓	✓						
18-month data collection^e^								✓	✓	✓				
24-month data collection^e^										✓	✓	✓		
Analysis							✓		✓		✓		✓	✓

^a^Q3 relates to July-September.

^b^Q4 relates to October-December.

^c^Q1 relates to January-March.

^d^Q2 relates to April-June.

^e^Primary outcomes assessed at 6 months (2025-Q2), 12 months (2025 Q4), 18 months (2026-Q2), 24 months (2026-Q4).

## Discussion

### Principal Findings

Effective models for scaling up digital care to maximize reach and impact at the population level remain elusive. This research trial will compare 2 models (clinician-initiated opt-in vs automated opt-out) of scaling up an evidence-based SMS text message program delivered directly to parents during the first 2000 days. While both scale-up models will offer families the same SMS text messaging program (HB4HNEKids), the “opt-in” arm focuses on providing CFHS clinicians with targeted implementation support strategies to onboard eligible families, and the “opt-out” arm represents an automated “hands-off” approach, whereby families are automatically connected to the program using health service data. Given that the opt-in model is expected to require greater clinician-parent interaction in relation to the program, we hypothesize that the opt-in model will be most effective for improving health behaviors, while the opt-out program is expected to achieve greater reach and representativeness of program participants and be more cost-effective.

mHealth interventions (eg, SMS text messages) delivered directly to parents’ mobile phones present an opportunity to revolutionize the delivery of universal health care across the first 2000 days. Key international agencies recommend universal preventative health care across the first 2000 days as a critical initiative for improving population health outcomes, and scaling up such initiatives have been considered critical for attaining the United Nations Sustainable Development Goal [14,44,45]. However, a review of 14 obesity prevention interventions delivered to infants and young children indicated that only 1 study had been formally evaluated at scale [46], suggesting that methods of achieving effective scale-up are significantly lacking for this demographic, limiting potential population health gains. This research seeks to bridge this evidence-practice gap by testing 2 methods of scaling up HB4HNEKids that have been co-designed and assessed as suitable for scale-up according to the New South Wales Guide to scaling up [47].

### Strengths and Limitations

A key strength of this research is the use of existing health service models, resources, and infrastructure to explore the most effective and efficient model of scaling up an evidence-based DHI that has huge potential to increase parental access and engagement with age-and-stage preventative health care. Despite this, research evaluating the reach and representativeness of program participants, a possible limitation of this research is the potential unintended widening of health inequalities. However, data suggest that people who are socially and economically disadvantaged, living in rural, regional, or remote areas, are Aboriginal or Torres Strait Islander, or are from culturally and linguistically diverse backgrounds, are among the highest users of digital technologies [48-50]. In addition, both scale-up models carry possible limitations in real-world implementation. For example, the impact of the clinician-initiated opt-in model relied heavily on clinician buy-in and engagement, and the automated opt-out model relies on having quality health system data; however, our evaluation is expected to highlight such problems. Furthermore, while efforts have been made to ensure a rigorous evaluation using multiple available streams of data sources, this evaluation will be limited to the quality of available data, with potential for significant missing data and reliance on self-reported data for secondary behavioral outcomes.

### Dissemination Plan

This research will address a critical policy and practice gap and generate real-world novel evidence to inform the delivery of other key DHIs to be implemented at scale, and as such, disseminating these findings is of critical importance. The findings from this study will be presented at international conferences and published in academic journals following the conclusion of data collection and analysis. The findings of this study will be further disseminated to health service agencies to ensure the learnings of this research can be applied broadly to local and national health service plans as well as other emerging digital health initiatives.

### Conclusions

Currently, there is limited evidence to guide the effective scale-up of mHealth interventions. Despite robust research evidence, effective mHealth interventions are rarely embedded within routine care, and no mHealth interventions have been implemented as usual care over the first 2000-day period. This research will be the first to compare the impact of “opt-in” versus “opt-out” approaches to better appraise their merits in scaling evidence-based programs and provide valuable insights into which model of care will facilitate the most equitable reach and uptake of digital health strategies.

## Appendix

Multimedia Appendix 1 SPIRIT checklist.

Multimedia Appendix 2 Logic model for the scale-up of HB4HNEKids.

## Abbreviations

AACTT: Action, Actor, Context, Target, Time
BCW: Behaviour Change Wheel
CFHS: Child and Family Health Service
CONSORT: Consolidated Standards of Reporting Trials
DHI: digital health intervention
ERIC: Expert Recommendations for Implementing Change
HB4HNEKids: Healthy Beginnings for Hunter New England Kids
HNELHD: Hunter New England Local Health District
mHealth: mobile health
RCT: randomized controlled trial
REDCap: Research Electronic Data capture
TDF: Theoretical Domains Framework

## Data Availability

This study is a study protocol, and no data are generated as part of this manuscript. Data sharing is not applicable to this article as no datasets were generated or analyzed during this study.
