# Supplementary material for: The Scale-Up of a Digital Health Intervention (Healthy Beginnings for HNEKids) Targeting the First 2000 Days: Protocol for a Randomized Controlled Trial
Source: JMIR Res Protoc. 2025 Dec 5;14:e81390. doi: 10.2196/81390 (PMC12717509; doi:10.2196/81390)
Supplement: Multimedia Appendix 2 [file resprot_v14i1e81390_app2.docx]

| Strategy | | Barrier | Capability, Opportunity, and Motivation Behavior system component (and TDF) [38] | Intervention function [38] | Behaviour Change Technique [39] | ERIC strategy [40] | Detailed explanation of strategy (according to the AACTT framework [41]) |
| --- | --- | --- | --- | --- | --- | --- | --- |
| **Preimplementation strategies – all services** | | | | | | | |
|  | Seek executive support/management endorsement | Lack of leadership [26] | Physical opportunity (Environmental context and resources) | Enablement | Restructuring the social environment | Involve executive boards | Gain executive support/endorsement for program scale-up from HNE Senior Leaders/ Managers prior to scale-up to ensure consistent program communication/awareness and support |
|  | Pilot program overview sessions | Clinician knowledge^#^  Clinician acceptability^#^  Resistance to change [26]  Professional skepticism [26]  Risk-benefit assessment [26] | Psychological capability (Knowledge) | Education  Training | Information about antecedents | Conduct educational outreach visits  Assess for readiness and identify barriers and facilitators | Increase awareness of the program among staff in HNE CFHS and address any concerns regarding different models being used for scale-up testing prior to group allocation. |
|  | Identify Key Contact | Inadequate relations across sectors^#^ [26]  Functional communication [26] | Physical opportunity (Environmental context and resources) | Enablement | Social support (practical) | Build a coalition | Establish effective communication channel with each CFHS sector prior to scale-up implementation activities to ensure consistent program communication/awareness |
|  | Ongoing communication with management | Lack of leadership[26] | Physical opportunity (Environmental context and resources) | Enablement | Restructuring the social environment | Inform local opinion leaders | Ongoing maintenance of CFHS management support for program activities and awareness of program outcomes to build support and recognition of the program |
|  | Brief introductory training | Clinician knowledge^#^  Clinician acceptability^#^  Clinician confidence^#^ | Psychological capability (Knowledge) | Education  Training | Information about health consequences | Make training dynamic | A brief (15 min) pre-recorded program introduction training video was made available, ensuring all CFHS staff can view at a convenient time, increase program knowledge and awareness, to better communicate with families.  A training/practice version of the onboarding system was made available. |
|  | Professional Taster Messages | Clinician knowledge^#^  Clinician acceptability^#^  Clinician confidence^#^ | Psychological capability (Knowledge)  Reflective Motivation (Intentions) | Education  Persuasion | Information about health consequences | Make training dynamic | CFHS staff will be invited to register (online) for a professional taster package, providing a sample (n=13) of the text messages received by families during the period of highest contact with the service (0-6 months). The daily messages are sent over a two-week period. Aimed to increase CFHS staff knowledge, confidence and acceptability of the program prior to program scale-up. |
| **Implementation strategies – clinician-initiated services only** | | | | | | | |
|  | Onboarding system | Capacity to register families^#^  Burden of work processes [26]  Excessive or unnecessary amount of data required [26]  User friendliness [26] | Physical Opportunity (Environmental context and resources) | Environmental restructuring  Enablement | Adding objects to the environment | Develop and organize quality monitoring systems | A simple online survey (REDCap) will be developed to facilitate ease of clinicians providing required data to trigger program commencement for families at time of initial contact. It will also function to allow clinicians to change a client’s mobile number or opt a client out of the program following subsequent client interactions. |
|  | Additional training support | Clinician acceptability^#^  Clinician confidence^#^  Perceived acceptability for diverse families^#^  Capacity to register families^#^  Lack of training [26] | Psychological capability (Knowledge)  Psychological capacity  (Behavioural regulation)  Physical capability (skills)  Automatic motivation (Emotion) | Education  Training  Persuasion  Enablement | Instructions on how to perform behaviour  Problem solving  Reduce negative emotions | Make training dynamic  Conduct education meetings/ conduct educational outreach visits | Based on site preference face-to-face or online training will be provided to CFHS staff immediately prior to program commencement that address knowledge and skills to support the following:   - Streamline enrolment processes that facilitate local tailoring - Ability to rapidly update family information to avoid delays - Address any concerns about program |
|  | Easy access link to onboarding system | Capacity to register families^#^  User friendliness [26] | Physical Opportunity (Environmental context and resources) | Environmental restructuring  Enablement | Restructuring the physical environment | Change physical structure and equipment | A link to the onboarding platform will be made easily accessible for clinicians by housing it on a Microsoft Teams shared channel, HB4HNEKids Collaboration Space. This also encourages CFHS staff to review other support materials including program “posts” that are saved on the HB4HNEKids Collaboration Space.  A link to the training/practice version of the onboarding system will also be available on the HB4HNEKids Collaboration Space. |
|  | Recordings of onboarding procedures | Clinician confidence^#^  Capacity to register families^#^  Staff issues, including stability and shortages [26]  User friendliness [26] | Physical capability (skills)  Physical Opportunity (Environmental context and resources) | Training  Environmental restructuring  Enablement | Instructions on how to perform behaviour  Adding objects to the environment | Develop educational materials  Model and simulate change | Recordings of onboarding pathways presented as part of the training will be made available via the HB4HNEKids Collaboration Space MS Teams Channel to provide a simple “how to” reference for clinicians that outlines the different onboarding pathways and can be updated as required. |
|  | Program script | Lack of time to explain program^#^ | Physical Opportunity (Environmental context and resources) | Enablement | Adding objects to the environment | Develop educational materials  Tailor strategies | Brief phone and text message script (explaining the program) developed for clinicians to use when onboarding families during first post birth contact via phone or text. |
|  | Program flyer | Remembering to offer program^#^ | Physical Opportunity (Environmental context and resources) | Environmental restructuring | Adding objects to the environment  Prompts/cues | Remind clinicians | Program flyers will be provided for display at the discretion of the service - in office area/s, family waiting rooms of each service, that will act as a prompt/reminder for clinicians to offer the program and for families to talk to clinician about program content. |
|  | Sector Champions | Different geographic and cultural features [26] | Physical Opportunity (Environmental context and resources)  Social Opportunity (Social influencers) | Environmental restructuring  Enablement  Modelling | Social support (practical) | Identify and prepare champions  Provide local technical assistance | Each sector will nominate at least one local/service level key contact prior to program implementation who will act as liaison between program staff and sector, to provide tailored feedback and support. |
|  | Nominated Support Officer | Functional communication [26]  Lack of established roles [26] | Physical Opportunity (Environmental context and resources) | Enablement | Social support (practical) | Identify and prepare champions  Facilitation | Each sector will be allocated a nominated Support Officer who will establish and maintain regular communication with the Sector Champion and will provide ongoing support for any queries or challenges experienced by the Sector. |
|  | Microsoft Teams shared support channel (HB4HNEKids Collaboration Space) | Clinician knowledge^#^  Clinician confidence^#^  Attitude of colleagues towards the technology [26] | Physical Opportunity (Environmental context and resources)  Social Opportunity (Social influencers) | Environmental restructuring  Enablement  Modelling | Restructuring the physical environment  Social support (practical) | Capture and share local knowledge  Centralise technical assistance  Distribute educational materials  Create a learning collaborative | Clinicians and administrative staff will be added to an online channel (MS Teams) that will contain video demonstrations on how to connect families, step by step infographic summarising how to onboard a family, FAQ. |
|  | Establish a Community of Practice | Clinician knowledge^#^  Clinician confidence^#^  Attitude of colleagues towards the technology [26] | Physical Opportunity (Environmental context and resources)  Social Opportunity (Social influencers)  Reflective motivation (learning from others experiences) | Enablement  Modelling | Social support (practical)  Social comparison | Capture and share local knowledge  Create a learning collaborative  Promote network weaving | An online Community of Practice will be established via the MS Teams online channel, connecting clinicians across sectors to share successes and provide advice/ expertise to overcome any challenges faced. |
|  | Quarterly reports | Limited information sharing [26]  Lack of incentives [26] | Psychological capability (Knowledge) | Education | Information about health consequences | Audit and provide feedback | Sectors will receive four tailored quarterly reports annually which will provide regular program updates and sector specific data that can be used by CFHS Managers to monitor performance.* |

“Onboarding” is defined as the process of recruiting, enrolling, and introducing the HB4HNEKids program to participants. *Automatic onboarding sites receive 4 generic, district wide quarterly reports annually. ^#^Qualitative interviews with CFHS staff during pilot phase of program (unpublished).

**References**

26. Borges do Nascimento IJ, Abdulazeem H, Vasanthan LT, Martinez EZ, Zucoloto ML, Østengaard L, et al. Barriers and facilitators to utilizing digital health technologies by healthcare professionals. npj Digital Medicine. 2023 2023/09/18;6(1):161. doi: 10.1038/s41746-023-00899-4.

38. Atkins L, Francis J, Islam R, O’Connor D, Patey A, Ivers N, et al. A guide to using the Theoretical Domains Framework of behaviour change to investigate implementation problems. Implementation science. 2017;12:1-18.

39. Human Behaviour Change Project. The Theory and Techniques Tool | Theory and Techniques Tool [Internet]. 2024; Available from: https://theoryandtechniquetool.humanbehaviourchange.org/tool.

40. Powell BJ, Waltz TJ, Chinman MJ, Damschroder LJ, Smith JL, Matthieu MM, et al. A refined compilation of implementation strategies: results from the Expert Recommendations for Implementing Change (ERIC) project. Implementation Science. 2015 2015/02/12;10(1):21. PMID: PMID: 25889199. doi: doi: 10.1186/s13012-015-0209-1.

41. Presseau J, McCleary N, Lorencatto F, Patey AM, Grimshaw JM, Francis JJ. Action, actor, context, target, time (AACTT): a framework for specifying behaviour. Implementation Science. 2019 2019/12/05;14(1):102. PMID: PMID: 31806037. doi: doi: 10.1186/s13012-019-0951-x.
